# What to Eat and Why

**Published:** 1911-07

**Authors:** 


**What to Eat and Why.** By **G. Carroll Smith,** M.D., of Boston, Mass. Octavo of 310 pages. Philadelphia and London: W. B. Saunders Company. 1911. (Cloth, $2.50 net.)

This is a sane and clever book upon a most important subject. The reader warms unconsciously, but agreeably, towards a writer when he learns from an early page that the book is not to be a compilation, but that the writer is to make an individual contribution. Of compilations we have too many. Of original contributions we can never have enough.

Evidently the author has been for many years a busy physician, and his attention has been called frequently to the role in etiology of improper habits of eating. From these observations have come convictions, and these convictions appear luminous and interesting in "What to Eat and Why."

The book is to be emphatically commended for its freedom from narrowness or extreme of enthusiasm; and for giving the reader a practical knowledge of the elements of nutrition, and of the role of the different varieties or kinds of food; of the need that meals should be attractive and have ample generosity and variety.

Again, the book will find a wide field of usefulness with practical busy physicians, who care for their patients, as contrasted with the over-worked specialists in diseases of the stomach who seem inclined to magnify their findings so as to at least make able patients pay well.

The book is written on the plane of practical work, free from the commercialising subtleties of either etiology, diagnosis or treatment. We believe that the field of dietotherapy is the most promising field in the entire realm of medical practice, and that books like Smith's "What to Eat and Why" will draw many members of our profession into that field to their great professional betterment, to the distinct advantage of their patients, and what is possibly of more importance than this—to the positive advancement of preventive medicine.

